# Comprehensive N-glycosylation profiling of recombinant spike S1 protein from the wild-type SARS-CoV-2 and its variants

**DOI:** 10.3389/fimmu.2025.1592142

**Published:** 2025-07-16

**Authors:** Yao Peng, Tian-Tian Tong, Qiu-Yu Deng, Lee-Fong Yau, Jia-Qi Qiu, Qing Zhao, Jia-Qi Wu, Zhi-Qiang Xin, Man-Ci Guan, Yue Li, Zhi-Hong Jiang, Hu-Dan Pan, Liang Liu, Jing-Rong Wang

**Affiliations:** ^1^ School of Pharmacy, Faculty of Medicine & Faculty of Chinese Medicine, Macau University of Science and Technology, Macao, Macao SAR, China; ^2^ Chinese Medicine Guangdong Laboratory, Guangdong-Macao In-Depth Cooperation Zone in Hengqin, Zhuhai, China; ^3^ State Key Laboratory of Traditional Chinese Medicine Syndrome, Guangzhou University of Chinese Medicine, The Second Affiliated Hospital of Guangzhou University of Chinese Medicine (Guangdong Provincial Hospital of Chinese Medicine), Guangzhou, China; ^4^ State Key Laboratory of Quality Research in Chinese Medicine, Macau Institute for Applied Research in Medicine and Health, Macau University of Science and Technology, Macao, Macao SAR, China

**Keywords:** SARS-CoV-2, spike protein, variants, N-glycans, chip LC/MS, N-glycopeptides

## Abstract

**Introduction:**

By 2024, COVID-19 has become endemic, with new variants contributing to its continued spread. The Spike protein forms trimers that bind to the ACE2 receptor on host cells, with the S1 subunit being a primary target for vaccines and antiviral treatments.

**Methods:**

Herein, we performed an in-depth analysis of the N-glycosylation of the recombinant Spike S1 protein (S1 protein) across the wild-type (WT) virus and its 5 variants, including Alpha, Beta, Gamma, Delta, and Lambda, by integrating ultrahigh-performance liquid chromatography coupled with quadrupole-time-of-flight mass spectrometry (UHPLC-Q-TOF MS) and unique TiO₂-PGC chip-based LC/MS techniques.

**Results:**

A total of 332 glycan structures arising from 180 compositions on the S1 and RBD regions were identified, revealing remarkable glycosylation diversity of the S1 protein. Complex glycan was shown to be the dominant structure across variants. Neutral N-glycans are mainly di-antennary with two fucosyl groups, while the majority of acidic N-glycans were multi-antennary with mono-fucosyl residues. In addition, sialic acid linkages of the N-glycans were extensively studied by utilizing ¹³C-labeled standards and specific enzymes for the first time, showing the existence of both α-2,3 and α-2,6 linkages across WT and five variants. It should be noted that the Lambda variant shows more complex α-2,3 and α-2,6-linked glycans in the RBD region, which may potentially enhance its glycan shield effect. Acetylated glycans, which were identified on S protein for the first time, were found to be fully fucosylated on the S1 region and sialylated on the RBD region across all variants. UHPLC-TOF MS analysis revealed unoccupied N-glycosylation sites in S1-Gamma (N657), S1-Delta (N61), and S1-Lambda (N17, N61, N657), with N17 and N61 showing low glycan occupancy (0%-3.4%), suggesting these sites may lack glycan shield protection.

**Discussion:**

This study provides a comprehensive N-glycosylation profile of the S1 protein across different variants, offering an essential structural basis for future vaccine development and research on viral functions.

## Highlights

- 27 acetylated N-glycans were identified on the Spike protein for the first time.
- 51 acidic N-glycans with α-2,3 or α-2,6 linkages were identified, and their distribution across WT and five variants (Alpha, Beta, Gamma, Delta, and Lambda) in the S1 and RBD regions was comprehensively mapped for the first time.
- Comprehensive glycosylation profiling was established for WT and five variants at the glycans and glycopeptide levels.

## 1 Introduction

Coronavirus disease 2019 (COVID-19) was a global pandemic caused by the severe acute respiratory syndrome coronavirus 2 (SARS-CoV-2). The virus expresses four structural proteins: spike (S) protein, membrane (M) protein, nucleocapsid (N) protein, and envelope (E) protein (). The largest S protein forms trimers that project from the virus surface (1–3), interacting with host cells via the angiotensin-converting enzyme 2 (ACE2) receptor (4–7). The S1 subunit of the S protein binds ACE2 and is a key target for the development of vaccine and antiviral therapies (8–11).

A distinguishing feature of the S protein is its extensive glycosylation, with 66 N-linked glycosylation sites per trimer (12–14). This glycan shield plays critical roles in protein folding, structural integrity (15, 16), immune evasion, and interaction with cellular factors (17, 18). Viral glycoproteins, including those on the S protein, can incorporate host-like glycans, potentially further obscuring the virus from the immune system (19–21). This shielding can impede antibody recognition and reduce vaccine efficacy (22, 23). Glycans also contribute to specific antibody recognition, as seen with the neutralizing antibody s309, which interacts with a glycan at N343 on the S protein (24). Glycosylation patterns are vital in the understanding of viral infectivity and immune evasion (25–27). Mass spectrometry methods like LC-MS/MS have become essential in analyzing these patterns, offering rapid and detailed insights into glycosylation sites. Most studies on SARS-CoV-2 S protein glycosylation were performed on the glycopeptide level. Studies have shown that differences in expression systems, methodologies, and glycan occupancy complicate the analysis of SARS-CoV-2 S protein glycosylation (28–32). Understanding the glycosylation microheterogeneity of the S protein is crucial, particularly as mutations in glycosylation sites can affect viral transmissibility and vaccine effectiveness (33). As new SARS-CoV-2 variants emerge, mutations in the S glycoprotein pose challenges to the effectiveness of vaccines and antibodies developed based on the original virus (34–36). SARS-CoV-2 variants have demonstrated resistance to current vaccines, emphasizing the need for the ongoing analysis of S protein glycosylation. Antibody-antigen recognition depends on protein-protein interactions (37–39), but glycans on S protein mask the antibody recognition site to prevent antibody-mediated neutralization (40, 41). Furthermore, it has been reported that mutation of N-linked glycosylation sites in the RBD of S1 protein (N331 and N343) dramatically reduced viral infectivity (42), indicating that N-glycosylation of S protein is essential for viral infectivity. Reports suggest that developing treatments for emerging viruses involves using surrogate phenotypes to assess effects, applying AI for protein folding, and high-throughput analysis of phenotype-related data for rapid response to viral threats (43). Targeting glycosylation using repurposed drugs could be a complementary strategy in controlling COVID-19 spread.

This study compares the N-glycosylation of recombinant S1 proteins from WT and different variants (Alpha, Beta, Gamma, Delta, and Lambda) expressed in HEK293 cells. We comprehensively analyzed the N-glycosylation in the S1 and RBD regions across WT and variants, focusing on both glycopeptides and N-glycans. Notably, linkages of acidic glycans were investigated by utilizing specific enzymes. Binding assays were also performed to explore the interaction of S1-antibody and ACE2 with different S1 protein samples. This study aims to discuss the N-glycosylation microheterogeneity across these variants and provide a foundation for future research on their correlation with viral infectivity and immune response. Insights gained from these studies could aid in developing improved vaccines and therapies, including monoclonal antibodies, to combat the spread of SARS-CoV-2 variants.

## 2 Materials and methods

### 2.1 Solvent, enzyme and recombinant proteins

LC-MS-grade acetonitrile (ACN) was purchased from J.T Baker (Avantor Performance Materials, LLC. Center Valley, PA, USA). LC-MS-grade formic acid (HCOOH), ammonium bicarbonate (ABC), iodoacetamide (IAA), dithiothreitol (DTT), and other chemicals were purchased from Sigma-Aldrich (St. Louis, MO, USA). Deionized water was prepared using a Milli-Q system (Millipore Ltd., Watford, UK). Sequencing grade-modified trypsin and chymotrypsin were purchased from Promega Corp. (Madison, WI, USA). PNGase F were purchased from New England Biolabs (Ipswich, MA, USA). α-2,3 sialidase (sialidase S) and α- 2,3,6,8,9 sialidase (sialidase A) were from Agilent (Santa Clara, CA, formerly Prozyme Hayward, CA).

Recombinant protein of wild-type SARS-CoV-2 spike subunit 1 (S1-WT, Cat. No. 40591-V08H; RBD-WT, Cat. No. 40592-V08H), Alpha S1 protein (S1-Alpha, Cat. No. 40591-V08H12; RBD-Alpha, Cat. No. 40592-V08H82), Beta S1 protein (S1-Beta, Cat. No. 40591- V08H15; RBD-Beta, Cat. No. 40592-V08H85), Gamma S1 protein (S1-Gamma, Cat. No. 40591-V08H14; RBD-Gamma, Cat. No. 40592-V08H86), Delta S1 protein (S1-Delta, Cat. No. 40591- V08H23; RBD-Delta, Cat. No. 40592-V08H90), Lambda S1 protein (S1- Lambda, Cat. No. 40591- V08H31; RBD- Lambda, Cat. No. 40592-V08H113) forms expressed in HEK293 cells were purchased from Sino Biological (Beijing, China). SARS-CoV-2 Spike Neutralizing Antibody (S1-antibody, Cat. No. 40591-MM43) and Angiotensin-converting enzyme 2 (ACE2, recombinant human ACE2 protein-mFc Tag, Cat. No. 10108-H05H) were obtained from Sino Biological (Beijing, China).

### 2.2 Protein digestion

S1 proteins were proteolyzed using an in-solution protease digestion protocol. Theoretical analysis of enzymatic sites showed that trypsin alone did not produce N-glycopeptides of appropriate length to cover all potential N-glycosites. The missing potential N-glycosites were recovered by introducing chymotrypsin digestion. Hence, we utilized a complementary trypsin and chymotrypsin digestion method to ensure that each N-glycosylation sequence from all recombinant S1 protein variants was covered by an N-glycopeptide of appropriate length to achieve optimal ionization and fragmentation. In brief, 100 μg of protein was dissolved in 100mM Tris-HCl, 10mM CaCl_2_ (pH 8.0) was denatured for 10 min at 95°C. After reduction by DTT (a final concentration of 5 mM) for 20 min at 50-60°C. The reduced protein mixture was cooled to room temperature, and IAA (a concentration of 15 mM) was added to alkylate the reduced cysteine residues for 15 min in the dark at room temperature. Chymotrypsin was added (enzyme/protein, 1:50, w/w), and the samples (at a final concentration of 1 μg/μL) were incubated at 25°C overnight. Trypsin was added (enzyme/protein, 1:50, w/w), and the samples (at a final concentration of 1 μg/μL) were incubated at 37°C overnight. The samples were then centrifuged at 14000 g for 15 min (4°C). The sample supernatant was collected and subjected to LC-MS analysis.

### 2.3 Release of N-glycans

N-glycans on the S1 proteins were cleaved using PNGase F. Briefly, 1 µL of PNGase F was added to the S1 protein solution, followed by incubation at 37°C for 16 hours. After enzymolysis, the cleaved N-glycans were loaded onto a C_18_ cartridge, and 1 mL of distilled water was used for elution, effectively removing excess proteins and enriching the N-glycans. The combined eluate was then concentrated by speed vacuum, and the resulting residues were reconstituted in 100 µL of distilled water. The solution was stored at -80°C until further analysis.

### 2.4 Protein desialylation

According to the operating instructions, 4 µL of 5x reaction buffer and 2 µL of sialidase S or sialidase A were added to the S1 protein stock solution. Subsequently, 1 µL of PNGase F was added, and the mixture was incubated at 37°C for 16 hours. This process removes sialic acid residues from N-glycans, forming α-2,3 and α-2,6 linked N-glycans, respectively.

### 2.5 UHPLC-Q-TOF-MS/MS method for glycopeptide profiling

SARS-CoV-2 S1 glycopeptides were analyzed using an Agilent 1290 Infinity UHPLC system coupled to an Agilent 6550 quadrupole-time-of-flight (Q-TOF) mass spectrometer (MS) with a dual Agilent jet stream electrospray ionization (Dual AJS-ESI) source (Agilent, Santa Clara, CA, USA). Chromatography was performed on an ACQUITY HPLC Peptide BEH C_18_ column (2.1×150 mm, 1.7 μm, 300Å). The mobile phase consisted of (A) 0.1% formic acid (FA) in water and (B) 0.1% formic acid (FA) in acetonitrile (ACN). A linear gradient was optimized as follows (flow rate, 0.3 mL/min): 0–5 min, 2% B; 5–35 min, 2% to 5% B; 35–87 min, 5% to 30% B, 87–92 min, 30% to 100% B, followed by washing with 95% B and equilibration with 2% B. The injection volume was 2 μL, and the column temperature was maintained at 40°C for each run. A typical run time was 92 min. The Dual AJS-ESI was performed in positive mode and the source parameters were as follows: dry gas (N_2_) temperature and flow rate was 250°C and 13 L/min, nebulizer pressure was 25 psi, sheath gas (N_2_) temperature and flow rate was 300°C and 11 L/min, capillary voltage was 4.5 kV, nozzle voltage was 300 V, fragmentor voltage was 380 V, skimmer voltage was 65 V. The acquisition mass range was m/z 100 to m/z 3200, and the scan rate was 2 spectra/s for both MS and MS/MS scan. A reference solution was nebulized for continuous calibration in positive mode using the reference masses of m/z 922.0098. The targeted MS/MS collision energy (CE) was set at three different values: 10–40 eV. The full scan and MS/MS data were processed using Agilent Mass Hunter Qualitative Analysis B.06.00 software.

### 2.6 N-glycan profiling by using TiO_2_-PGC chip-Q-TOF MS

N-glycans profiling of the S1 protein was conducted using an Agilent 1200 Series HPLC-Chip system, interfaced with an Agilent 6546 Q-TOF-MS. The TiO_2_-PGC chip was continuously operated in forward flush mode. For the capillary pump, the mobile phase consisted of 0.6% acetic acid, 2% formic acid (FA), and 2% acetonitrile (ACN) in water transferred to the enrichment column at a flow rate of 3 µL/min. The nanoflow pump utilized a mobile phase of 1% FA in water (A) and ACN (B) for neutral N-glycans. The gradient for the separation process was maintained at a flow rate of 0.5 µL/min as follows: 0~6 minutes, 5% B; 6~16 minutes, 5% to 60% B; 16.1~19 minutes, 80% B. The sialylated N-glycans were then eluted by injecting 5 µl of 0.5% ammonia and switching to a mobile phase optimized for acidic N-glycans. Mobile phase A was 0.5% FA in water adjusted to pH 3 with ammonia, and mobile phase B was 1% FA in ACN. The flow rate was 0.5 µl min−1, and the gradient was 0~1 min, 5% B; 1~11min, 5–60% B; 11~14min, 80% B. The N-glycan structures were elucidated using high-resolution MS and MS/MS data obtained in the positive mode of the Q-TOF MS. The dry gas (N_2_) temperature was set to 200°C, with a flow rate of 8 L/min. MS spectra were acquired in positive ion mode over the mass range of 500–3000 m/z, with the data acquisition rate of 1 spectrum per second. MS/MS experiments covered the mass range from 200 to 3000 m/z in targeted MS/MS mode with the acquisition rates of 2 spectra per second for MS and 3 spectra per second for MS/MS. The collision energy (CE) was set between 10–30 eV.

### 2.7 Data analysis

All MS and MS/MS data were analyzed using Agilent Mass Hunter Qualitative Analysis B.06.00 software. The find-by-formula (FBF) algorithm was used to identify glycopeptides from the SARS-CoV-2 S1 protein and its variants. Key parameters included a maximum of five possible matches per formula, ± 15 ppm mass tolerance, and matching for proton adducts (+H) with charge states of 1-5. Only results with an overall score above 70 were included. Glycopeptide CID fragmentation data provided valuable information for assigning glycan compositions, including oxonium ions such as m/z 163 [Hex + H] ^+^, m/z 204 [HexNAc + H] ^+^, and m/z 366 [Hex-HexNAc + H] ^+^ and Y- or b-type ions from the peptide moiety. Therefore, glycopeptide compositions were assigned with the aid of these MS/MS data. Each protease digest (trypsin, chymotrypsin) was searched with appropriate digestion parameters, allowing for two missed cleavages. N-glycans were classified based on composition, starting with the core structure Hex (3) HexNAc(2). High-mannose glycans were characterized as Hex (4−12) HexNAc(2), while hybrid N-glycans were categorized based on additional components, such as fucose and sialic acid. Complex-type N-glycans were classified by the number of antennary structures, sialylation, and fucosylation levels. These were further divided into mono-, di-, tri-, and tetra-antennary forms. Glycans were classified as sialylated or fucosylated if they contained at least one sialic acid or fucose residue, respectively.

### 2.8 Binding kinetic assay by using bio-layer interferometry

The Octet^®^ RED96 system (FortéBio, Fremont, CA, USA) was utilized in this study as well as the Anti-Mouse IgG Fc Capture (AMC) biosensors (FortéBio, CA, USA). For the binding kinetic assay of S1-antibody or ACE2 (Sino Biological, Beijing, China) with different recombinant S1 proteins, the S1-antibody of 15 μg/mL or ACE2 of 15 μg/mL buffered in kinetic buffer (PBS buffer supplemented with 0.02% Tween 20, pH 7.4) was immobilized onto AMC biosensors, and then incubated with two-fold serial dilutions of S1 protein (the initial concentration was 6.67 μg/mL) in kinetic buffer. The AMC biosensors were equilibrated in kinetics buffer for 10 min at room temperature before data acquisition. All experiments were performed at 30°C and 1000 rpm according to the protocol from the manufacturer. Briefly, AMC biosensors were dipped in 200 μL of S1-antibody solution or ACE2 solution for the loading step. The biosensors loaded with S1-antibody (or ACE2) were then sampled with S1 protein at various concentrations in kinetic buffer to obtain the association curve. After association, the biosensors were dipped back into kinetic buffer to obtain the dissociation curve. The background signal was subtracted from all samples by dipping a S1-antibody (or ACE2) immobilized biosensor in the blank kinetic buffer. After subtraction, binding kinetic constants were determined from at least 5 concentrations of S1 protein. By fitting the curves to a 1:1 binding model using Octet Data Analysis Software v11.1 (FortéBio, Torrance, CA, USA), the resulting equilibrium dissociation constant (KD) values were calculated for the interaction.

## 3 Results

### 3.1 In-depth profiling of N-glycans on SARS-CoV-2 S1 protein

By employing a unique TiO_2_-PGC chip-based nano LC separation technique, coupled with time-of-flight mass spectrometry, the glycans on S1 protein of WT and five variants were analyzed extensively in the current study. For the identification, a personal database with 1149 N-glycans was established. The FBF algorithm in Mass Hunter software was used to match the molecular formula, m/z, and RT of each glycan composition. Characteristic ion fragments were then obtained through MS/MS target data analysis. Chromatographic behavior and MS characteristics of N-glycans, including retention time (RT), mass-to-charge ratio (m/z), and MS^2^ fragmentation were summarized (**Supplementary Table S1**). Notably, N-glycans with specific glycosylation modifications, such as core fucosylation, were identified by characteristic ion fragments with m/z of 350.14 and 553.21. Similarly, N-glycans with sialylation exhibited distinct ion fragments with mass-to-charge ratios of 274.08, 292.09, and 657.22, aiding in their structural confirmation.

As a result, a total of 332 N-glycan structures arising from 180 N-glycan compositions were characterized, including 112 neutral glycans derived from 66 compositions and 210 acidic glycans arising from 114 compositions.

The glycan profiling showed significant structural difference between neutral and acidic N-glycans. For instance, of the 66 neutral N-glycans identified, most species had two fucosyl groups, while on 115 acidic N-glycans, mono-fucosylation was dominant. Structurally, neutral N-glycans were typically di-antennary, whereas acidic N-glycans were multi-antennary.

**Figure 1:**
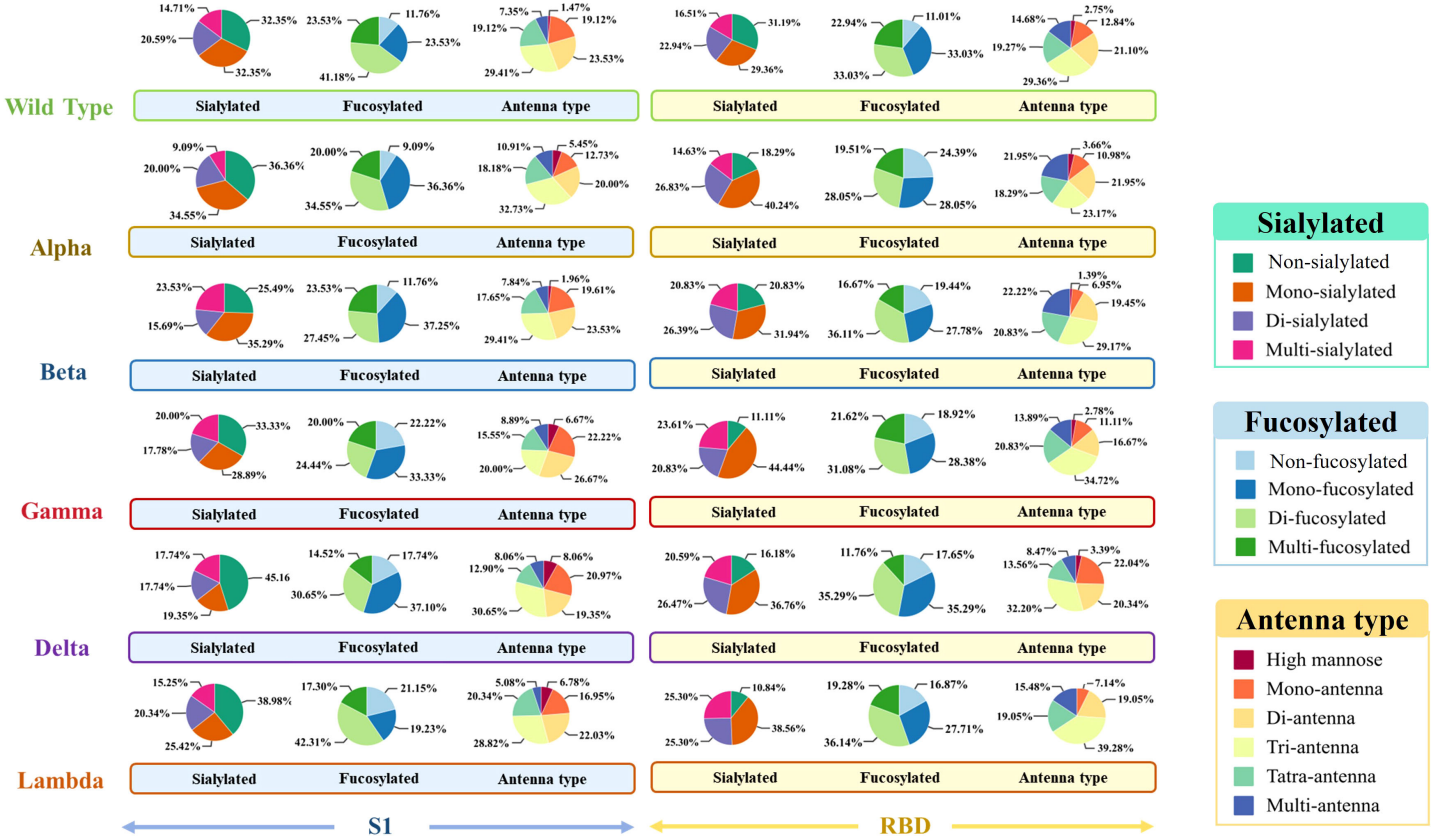
The glycosylation profiles of N-glycans in the S1 and RBD regions of WT and five variants. Differences in sialylation, fucosylation, and structure characteristics of N-glycans in WT and five variants, including Alpha, Beta, Gamma, Delta, and Lambda.

To explore the differences across WT and variants, the N-glycan distribution in the S1 and RBD regions of WT, Alpha, Beta, Gamma, Delta, and Lambda was analyzed (**Figure 1**). The results suggested regional-specific variation of glycans across WT and variants. In the S1 region, WT and five variants exhibited a general high fucosylation level (> 70%), and the N-glycans structures were predominantly di-fucosylated. The S1-Alpha showed the highest fucosylation level among all variants at 91.9% (9.09% non-fucosylation), while the S1-Gamma and S1-Lambda showed comparatively lower fucosylation levels, ranging from 77.8% to 78.9%, respectively. However, fucosylation on the RBD region showed opposite distribution across variants, as exemplified by the lowest fucosylation observed for the Alpha variant (75.7%), while the Lambda variants had higher levels at 83.1%. Moreover, sialylation was observed across all variants, with mono-sialylated glycans occupying the predominant proportion. The Delta variant exhibited the lowest sialylation level in the S1 region, with only 54.9%. However, in the RBD region, the Delta variant presents a high sialylation level of 83.9%. This highlights special glycosylation modifications across variants, with notable differences between regions.

**Figure 2:**
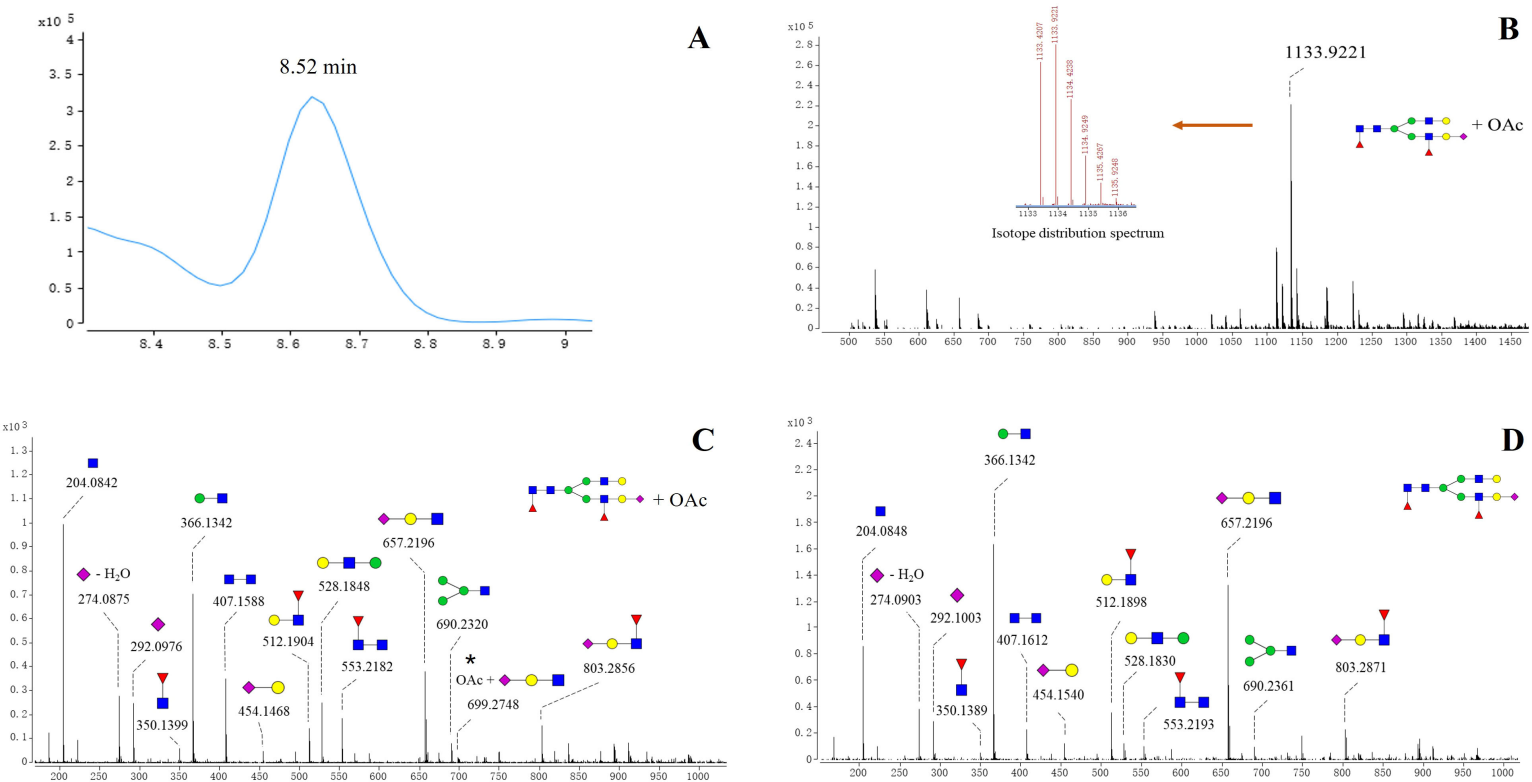
Structures of acetylated N-glycan (5_4_2_1+ OAc) identified on spike protein. **(A)** EIC spectrum of 5_4_2_1 + OAc. **(B)** Positive mode CID MS spectrum of 5_4_2_1(A) + OAc. The m/z value of the molecular ion peak for 5_4_2_1 (A)+OAc is 1133.9221, and its isotopic distribution aligns with the predicted pattern, further verifying the MS spectrum of 5_4_2_1 (A)+OAc. **(C)** Positive mode CID MS/MS spectrum of 5_4_2_1 + OAc. **(D)** Positive mode CID MS/MS spectrum N-glycan 5_4_2_1.

Notably, acetylated N-glycans were identified on the S1 protein for the first time. The characterization of acetylated N-glycans was exemplified herein by 5_4_2_1+OAc (**Figure 2**). The ***m/z*** value, charge state, molecular formula, and RT of the 5_4_2_1+OAc were confirmed through the FBF algorithm. The extracted ion chromatogram (EIC) of 5_4_2_1+OAc showed a compound at RT 8.52 min (**Figure 2A**). The mass spectrum for 5_4_2_1+OAc (**Figure 2B**) displayed molecular ion peaks at m/z value of 1133.9221, and zooming of this spectrum confirmed that the isotopic distribution matched the expected pattern. The MS/MS spectrum of the non-acetylated N-glycan 5_4_2_1 (**Figures 2C, D**) indicated the absence of key ion fragments related to acetylation, supporting the presence of acetylated N-glycans on the S1 protein. In total, 27 N-acetylated N-glycans were identified, and their m/z values, RT, mass tolerance, and characteristic fragment ions were documented (**Supplementary Table S2**). In the WT and five variants, the acetylated N-glycans in the S1 region were predominantly fucosylated, with di-fucosylated and di-sialylated N-glycans being the most common glycosylation modifications. Structurally, acetylated N-glycans exhibited various configurations, including mono-, bi-, tri-, tetra-, and multi-antennary forms, with tri-antennary structures being the most prevalent. However, the RBD region contained non-fucosylated glycans (4_5_0_2+OAc and 4_6_0_2+OAc) and also displayed a more complex and diverse pattern of sialylation, distinguishing it from the acetylation characteristics observed in the S1 region (**Supplementary Table S3**). Further analysis of acetylated glycans across various variants revealed higher acetylation of glycans in the S1 region than RBD region of WT, Alpha, and Beta variants (**Supplementary Table S3**) Further analysis revealed that acetylated glycans, unlike other N-glycans, were not predominantly concentrated in the RBD region across variants but exhibited greater accumulation in the S1 region.

### 3.2 Distribution of α-2,3 and α-2,6 linked sialylated N-glycans on SARS-CoV-2 S1 protein

Sialylation typically exists in two prominent linkages: α2,3 and α2,6, catalyzed by sialyltransferases such as ST6Gal1, ST3Gal3, ST3Gal4, and ST3Gal6. Influenza viruses rely on sialic acid receptors to enter host cells, and the glycosidic bonds linking terminal sialic acids—specifically α-2,3 and α-2,6 linkages— were widely recognized as critical determinants for the cross-species transmission of these viruses (44). ST3GAL2 resists viral infection by downregulating pro-inflammatory cytokines (IL-6, IL-18, IL-1β, IFN-β, TNF-α) and upregulating anti-inflammatory cytokines (IL-4, IL-10, IL-13) (45). ST6Gal1 sialyltransferase also enhances Newcastle disease virus binding and cytopathic effects (46). TiO_2_-PGC chips offer excellent separation of N-glycan isomers. Sialic N-glycans with α-2,3 or α-2,6 linkages show distinct RT on the TiO_2_-PGC chip, thus allowing for the differentiation of glycans with different sialic linkages. This part provides the first comprehensive analysis of the distribution of acidic glycans with α-2,3 and α-2,6 linkages across various regions and variants of six prevalent variants during the pandemic. N-glycans with α-2,3 linkages are the predominant species in both the S1 and RBD regions, while glycans with α-2,3 and α-2,6 linkages exhibit extensive fucosylation and distinct profiles across all WT and five variants. To confirm the separation performance of PGC chip for sialylated glycan isomers, ^13^C-labeled G2FS (5_4_1_1) standards with α-2,3 and α-2,6 linkages were analyzed simultaneously on HPLC chip-Q-TOF MS.

**Figure 3:**
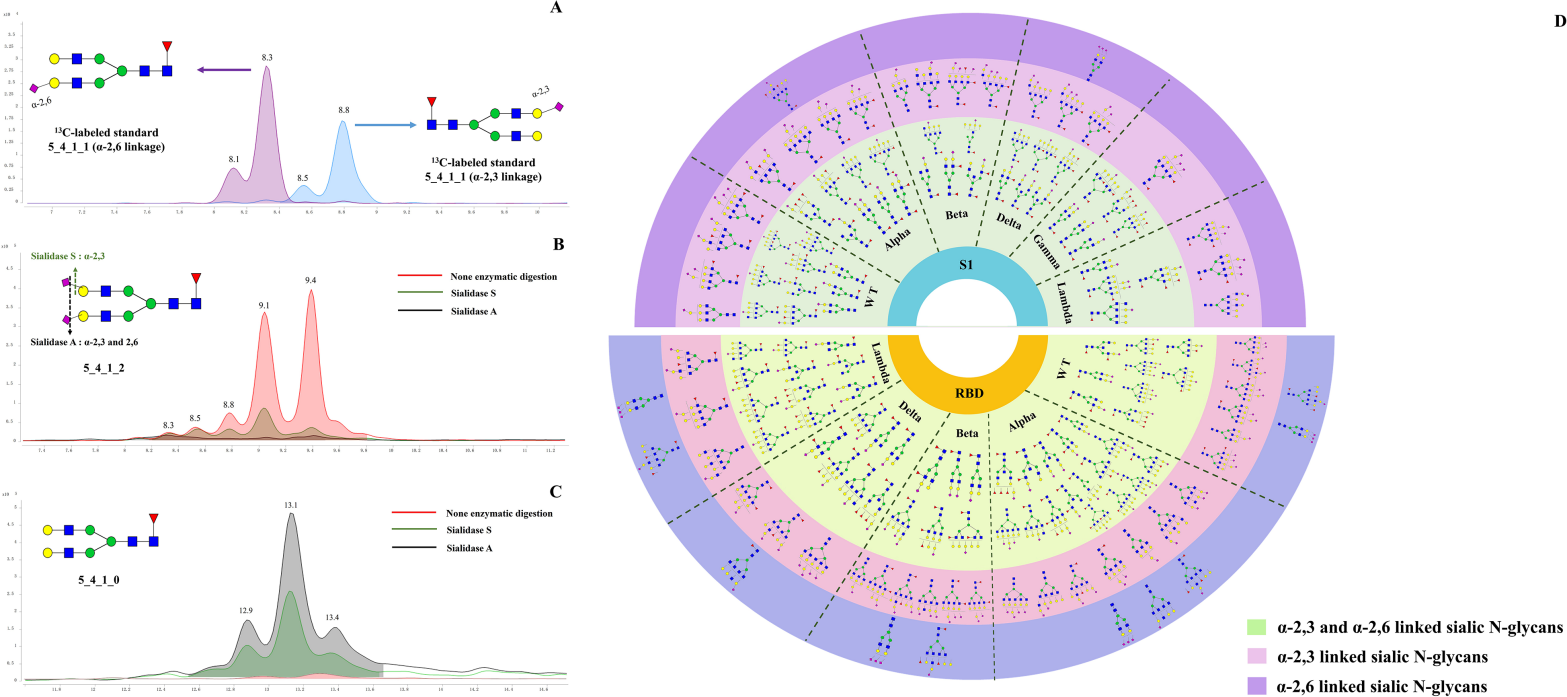
Determination of acidic N-glycans with α 2,3 and 2,6 linked by using TiO_2_-PGC chips. **(A)** ^13^C-G2FS (5_4_1_1) standards with α 2,3- and 2,6-linkages were analyzed using PGC-TiO_2_ chips. The sialic acid isomers linked to ^13^C-G2FS (5_4_1_1) were separated on the TiO_2_-PGC chip, with α 2,6-linked sialic acid N-glycans eluting earlier than α 2,3-linked. **(B)** The EIC overlapped spectrums of 5_4_1_2 following digested by sialidase A and sialidase S enzymes. **(C)** The EIC overlapped spectrums of 5_4_1_0 following digested by sialidase A and sialidase S enzymes. **(D)** Distribution of α-2,3 and α-2,6 linked sialylated N-glycans on WT and five variants.

As shown in **Figure 3A**, the ^13^C-labeled biantennary acidic glycan standards 5_4_1_1 (α-2,3 linkage and α-2,6 linkage) exhibited multiple isomeric peaks, with the α-2,6 linked 5_4_1_1 eluting earlier than the α-2,3 linked, demonstrating the method’s ability to effectively differentiate between these two sialic acid-linked glycans. To confirm the isomeric linkage, enzymatic hydrolysis with sialidase S and sialidase A was further performed. Sialidase S specifically hydrolyzes α-2,3-linked sialic acid residues, while sialidase A cleaves all sialic acid residues with either α-2,3 or α-2,6 linkages. By comparing the abundance of sialylated N-glycans before and after enzymatic treatment, the linkage of the glycans can be confirmed. After sialidase S treatment, the abundance of acidic glycan 5_4_1_2 appeared at 8.8, 9.1, and 9.4 minutes significantly decreased, showing that these signals arise from α-2,3-linked sialic N-glycans. In contrast, peaks at 8.3 and 8.5 min remained unchanged upon sialidase S treatment, but decreased with sialidase A treatment, suggesting α-2,6 linkages in these glycans (**Figure 3B**). Assignment of the linkage were further evidenced by the observation of increased abundance of the corresponding neutral glycan (5_4_1_0) following enzymatic digestion. (**Figure 3C**). Specifically, the abundance of 5_4_1_0 increased after enzymatic digestion with both Sialidase A and Sialidase S, compared to the non-digested condition. Among them, the highest level of 5_4_1_0 was observed in the sample treated with Sialidase A, followed by that treated with Sialidase S. By employing this isomer-specific chip LC method, 47 compositions with α-2,3 linkages and 26 compositions with α-2,6 linkages were identified, including 85 α-2,3-linked N-glycan structures and 38 α-2,6-linked N-glycan structures (**Supplementary Table S4**).

Using strategies described above, linkages of 51 acidic N-glycans were characterized on the S1 proteins of WT and five variants (**Supplementary Table S4**). It seems that N-glycans featured by three or four antennas tend to be capped with α-2,6-linked sialic acid rather than α-2,3-linked sialic acid (**Figure 3A**), while those N-glycans with more simple structures tends to be sialylated with both linkages. The results showed that bi-antennary and bisecting types of N-glycans (e.g., 4_4_1_1, 5_4_0_1, 4_5_2_1, 5_5_1_2, and 5_5_2_1) preferred to be sialylated with both α-2,3 and α-2,6 linkage types. Notably, the distribution of α-2,3 and α-2,6 linked N-glycans differed between the S1 and RBD regions. α-2,3 linked N-glycans were found to be dominant species across WT and five variants, both in the S1 and RBD regions (**Figure 3D**). However, α-2,6 linked N-glycans show more abundant in the RBD region. Structurally, N-glycans with α-2,3 or α-2,6 linkages in the RBD region generally exhibit more complex glycan structures, particularly tri- or tetra-antenna structures, whereas those in the S1 region were characterized by simpler branching structures.

Furthermore, the distribution of α-2,3 and α-2,6 linked glycans also differs among the variants. For the Alpha, Beta, Delta, and Gamma variants, α-2,3 and α-2,6 linked glycans were predominantly associated with multi-fucosylated N-glycans and exhibit di or tetra-antenna structures. Differently, the S1-Lambda predominantly features fucosylated tri- or tetra-antennary α-2,3 and α-2,6 linked glycans, with simpler modification, characterized by mono- or di-fucosylation and sialylation, lacking complex sialic acid modifications and extensive fucosylation seen in other variants.

### 3.3 Characterization of site-specific glycosylation on SARS-CoV-2 S1 protein

In this study, we used UHPLC-Q-TOF to identify N-glycopeptides within the S1 region of the S1 protein. We analyzed the secondary mass spectrometry fragments of each glycopeptide, using specific fragment ions to confirm the presence of these N-glycopeptides (**Supplementary Table S5**). The S1-WT has 13 possible N-glycosites, including N17, N61, N74, N122, N149, N165, N234, N282, N331, N343, N603, N616, and N657 (**Supplementary Figure S1**). N331 and N343 N-glycosites were located in the RBD domain. N-glycan occupancy is a crucial parameter to consider in immunogen design.

#### 3.3.1 Site-specific glycopeptide diversity across different variants

**Figure 4:**
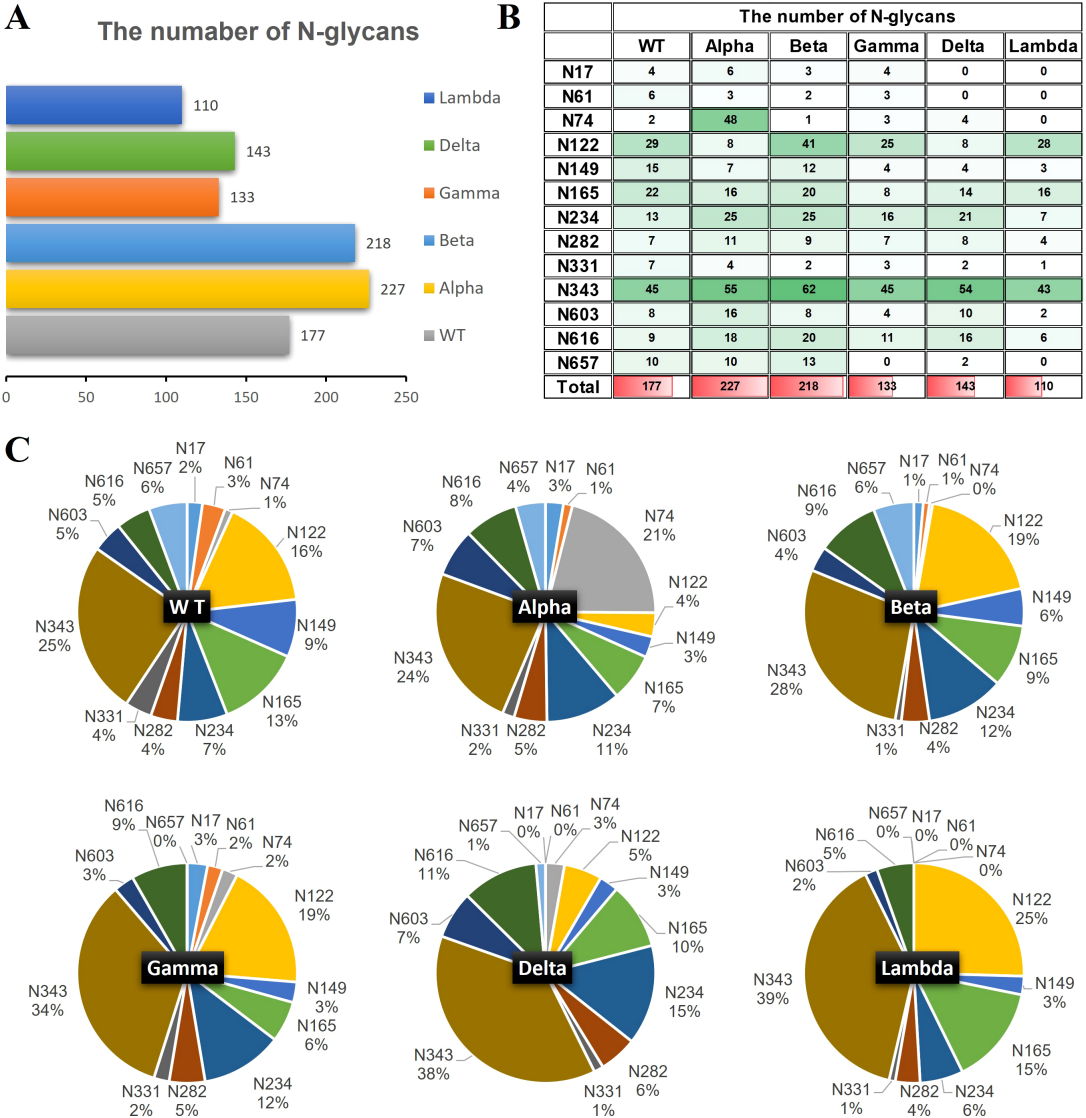
The number of site-specific N-glycosylation profile of S1-WT and five variants. **(A)** The bar chart showed the total number of identified N-glycopeptides of S1 proteins. **(B)** The number of N-glycans identified at the 13 glycosites. **(C)** The pie chart showed the proportion of N-glycans at each N-glycosylation site of S1 protein.

The quantities of N-glycopeptides at specific sites across the WT and Alpha, Beta, Delta, Gamma, and Lambda variants were analyzed and summarized. 177 N-glycopeptides (including 116 N-glycan compositions) were identified at 13 predicted N-glycosylation sites of the S1-WT (**Figure 4A**). 227 N-glycopeptides (including 146 N-glycan compositions) at 13 N-glycosites of S1-Alpha were identified. 218 N-glycopeptides (including 144 N-glycan compositions) across the N-glycosylation sites of S1-Beta were identified. 133 N-glycopeptides (including 93 N-glycan compositions) at 12 N-glycosites of S1-Gamma were identified. 143 N-glycopeptides (including 102 N-glycan compositions) at 11 N-glycosites of S1-Delta were identified. 110 N-glycopeptides (including 83 N-glycan compositions) at 9 N-glycosites of S1-Lambda were identified.

The data revealed extensive micro-heterogeneity across glycosites, with the number of identified N-glycans at each site ranging from 2 to 45 (**Figure 4B**). Glycosites such as N17, N61, N74, N282, N331, N603, N616, and N657 exhibited less glycan variety, while N122, N149, N165, N234, and N343 showed greater diversity (**Supplementary Table S6**). Specifically, the N343 site in the RBD domain of S1-WT has the highest number of N-glycans (~25%), with N122 also exhibiting a high proportion (~16%). Interestingly, the N343 site consistently holds the highest proportion of N-glycans across five variants, with ~24% in S1-Alpha, ~28% in S1-Beta, ~34% in S1-Gamma, and ~38% in S1-Delta, all of which significantly surpass that of other sites (**Figures 4B, C**). However, the N657 site in S1-Gamma was found to be unglycosylated, and it remains unclear whether this is related to the nearby H655Y mutation. In S1-Delta, the T19R substitution eliminates glycosylation at the N17 site, and no N-glycans were detected at the N61 site, except for the N17 site (**Figure 4C**). Similarly, in S1-Lambda, the T76I mutation abolished N-glycosylation at N74, and three sites (N17, N61, and N657) were completely unoccupied by N-glycopeptides, apart from N74.

#### 3.3.2 Site-specific glycan types and antennary patterns across different variants

**Figure 5:**
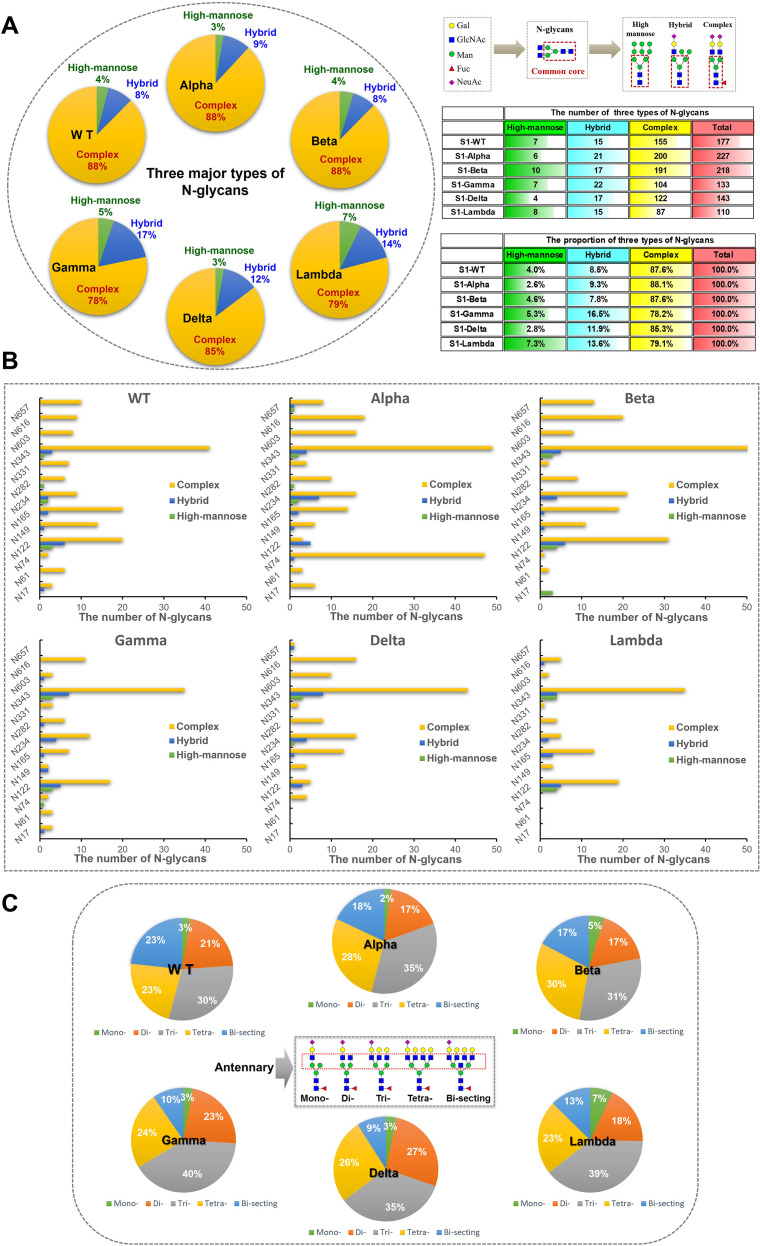
Classification results of different types and numbers of N-glycans on S1 proteins. **(A)** N-glycans were assigned to glycosylation sites of the S1 protein and the counts for each of the three types of N-glycans (high-mannose, hybrid, and complex) were summed and visualized as pie charts. N-glycosylation sites of recombinant S1 proteins were predominantly modified by complex N-glycans. **(B)** The bar graph provides additional detail about the site-specific N-glycans were grouped by broad N-glycan type. **(C)** The pie chart shows the proportion of N-glycans were grouped by antennary complex- type N-glycan. According to the numbers of antennary, N-glycans can be classified into mono-, di-, tri-, and tetra-antennary. The tri- and tetra-antennary account for the majority of identified complex-type N-glycans.

The glycan compositions at each site were classified by subtype and visualized in pie and bar charts (**Figures 5A, B**). In S1-WT, 116 N-glycan compositions were predominantly complex-type (~88%), with minor hybrid (~8%) and high-mannose (~4%) N-glycans. Eleven sites had over 75% complex-type N-glycans, with six sites (N61, N74, N331, N603, N616, and N657) fully occupied by complex-type N-glycans. The N122 and N234 sites had lower complex-type occupancy (~69%) (**Figure 5B**). In S1-Alpha and S1-Beta, ~ 88% of detected N-glycans were complex-type. Five sites in S1-Alpha (N17, N61, N331, N603, and N616) and seven sites in S1-Beta (N61, N74, N282, N331, N603, N616, and N657) were fully occupied by complex-type N-glycans, while the N17 site in S1-Beta was fully occupied by high-mannose N-glycans (**Figure 5B**). In S1-Delta, ~ 85% of N-glycosylation sequons were complex-type, with six sites (N74, N149, N282, N331, N603, and N616) fully occupied by complex-type N-glycans (**Figures 5A, B**). In S1-Gamma and S1-Lambda, complex-type N-glycans were reduced to ~78% and ~79%, respectively. In S1-Gamma, three sites (N61, N331, and N616) were fully occupied by complex-type N-glycans, with hybrid N-glycans increasing to ~17%, while the remaining sites contained high-mannose. In S1-Lambda, four sites (N149, N282, N331, and N603) were fully occupied by complex-type N-glycans (**Figure 5B**).

In S1-WT, tri-antennary N-glycans (~30%) were the most abundant complex-type N-glycans, followed by tetra-antennary (~23%), bi-secting (~23%), di-antennary (~21%), and mono-antennary (~3%) types (**Figure 5C**). In S1-Alpha, tri-antennary (~35%) and tetra-antennary (~23%) N-glycans were more prevalent, while bi-secting (~18%), di-antennary (~17%), and mono-antennary (~2%) types were less common. S1-Beta also showed a high proportion of tri-antennary (~31%) and tetra-antennary (~30%) N-glycans. In S1-Gamma, tri-antennary N-glycans (~40%) were more prominent than in other variants. The S1-Delta variant featured a higher proportion of tri-antennary (~35%) and di-antennary (~27%) N-glycans, with lower amounts of tetra-antennary (~26%), bi-secting (~9%), and mono-antennary (~3%) types. Finally, in S1-Lambda, tri-antennary (~39%) and tetra-antennary (~23%) N-glycans were the dominant forms (**Figure 5C**).

#### 3.3.3 Site-specific glycosylation modifications across different variants

**Figure 6:**
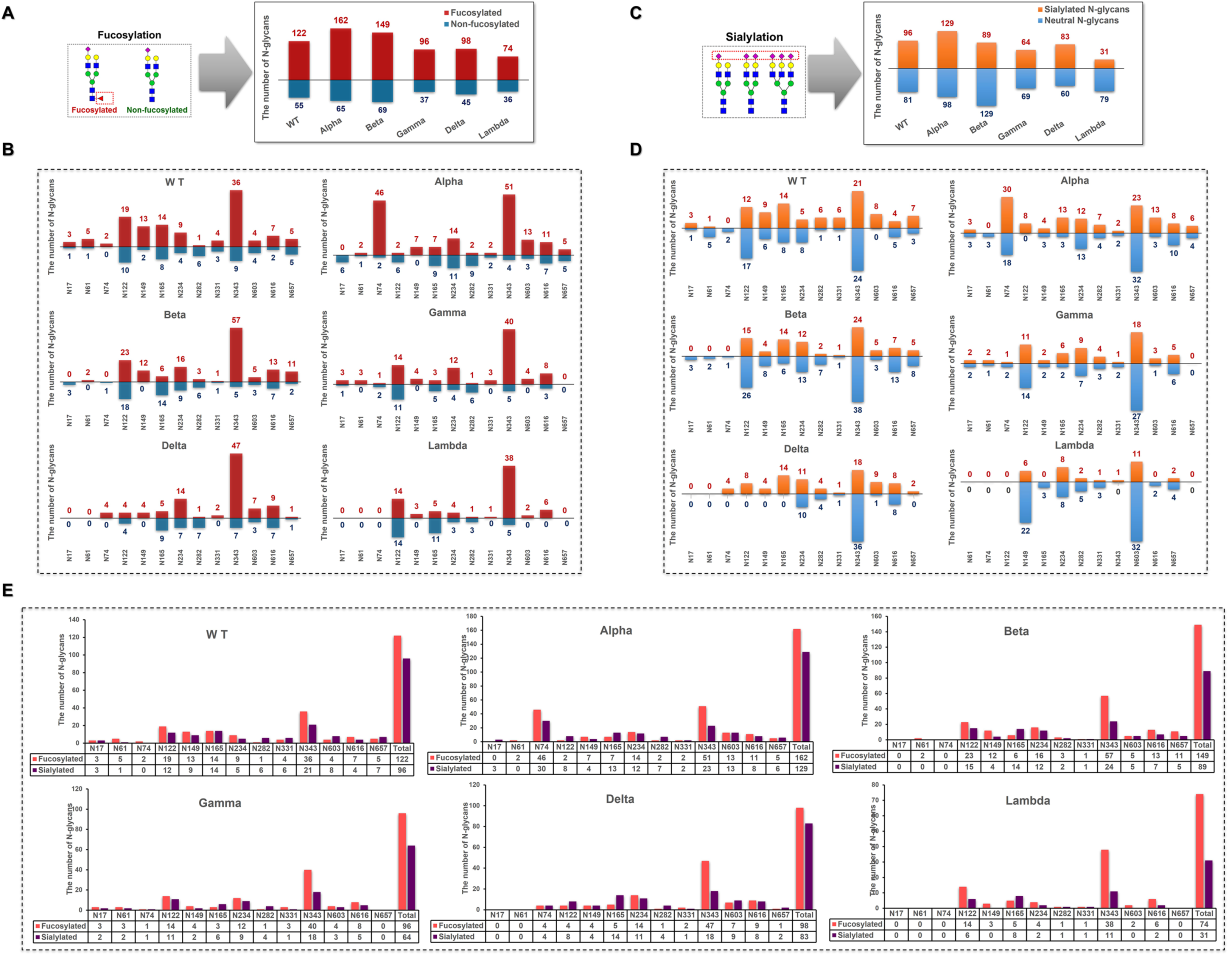
Distribution of fucosylated and sialylated N-glycans at glycosites within the sequence range of the S1 subunit. **(A)** Comparison of the number of fucosylated N-glycopeptides identified in different S1 protein samples. **(B)** The number of N-glycan compositions identified per glycosite was grouped by the presence or absence of fucose. **(C)** Comparison of the number of sialylated N-glycopeptides identified in different S1 protein samples. **(D)** The number of N-glycan compositions identified per glycosite was grouped by the presence or absence of sialic acid. **(E)** Comparison of the number of site-specific fucosylated and sialylated N-glycans in different S1 protein samples.

In S1-WT, 69% of N-glycans were fucosylated (**Figure 6A**), with N74 being fully fucosylated (~100%) and over 80% of N-glycans at N61 (~83%), N149 (~87%), and N343 (~80%) sites bearing fucose residues (**Figure 6B**). However, only ~14% of fucosylated N-glycans were detected at N282. Additionally, 54% of N-glycans contained sialic acid, with N17, N282, N331, and N603 sites showing high sialylation (>75%), especially N603, which was fully sialylated (**Figures 6C, D**). The N74 site was occupied by both highly fucosylated (~100%) and sialylated N-glycans. In contrast, most N-glycans at N282 were sialylated (~86%) and lacked fucose (**Figure 6E**).

In S1-Alpha, 71% of N-glycans were fucosylated, and 57% were sialylated. N149 was fully fucosylated (~100%), while N17 contained no fucosylated N-glycans (**Figure 6B**). The N122 site was exclusively occupied by sialylated N-glycans (**Figure 6D**). The highest numbers of both fucosylated and sialylated complex-type N-glycans were found at N74 and N343 sites (**Figure 6E**).

In S1-Beta, 68% of N-glycans were fucosylated and 41% were sialylated. N61 and N149 were fully fucosylated, while N17 and N74 sites contained only non-fucosylated N-glycans (**Figure 6B**). Additionally, N17, N61, and N74 sites were fully non-sialylated (**Figure 6D**). The highest numbers of both fucosylated and sialylated complex-type N-glycans were found at N122 and N343 sites (**Figure 6E**).

In S1-Gamma, 72% of N-glycans were fucosylated, which was the highest among the five variants (**Figure 6A**), with N61, N149, N331, and N603 sites fully fucosylated (**Figure 6B**). On average, 48% of N-glycans were sialylated, with N165 and N603 showing ~75% sialylation (**Figures 6C, D**).

In S1-Delta, 69% of N-glycans were fucosylated, with N74, N149, and N331 sites fully fucosylated (**Figures 6A, B**). Additionally, 58% of N-glycans were sialylated, with N74, N122, N149, N165, and N657 sites completely occupied by sialylated N-glycans (**Figures 6C, D**).

In S1-Lambda, 67% of N-glycans were fucosylated, with N149, N331, N603, and N616 sites fully fucosylated (**Figures 6A, B**). However, only 28% of N-glycans were sialylated, with N149 and N603 sites fully sialylated (**Figures 6C, D**).

### 3.4 Assessment of the binding affinity of S1-antibody and ACE2 to different S1 proteins

**Figure 7:**
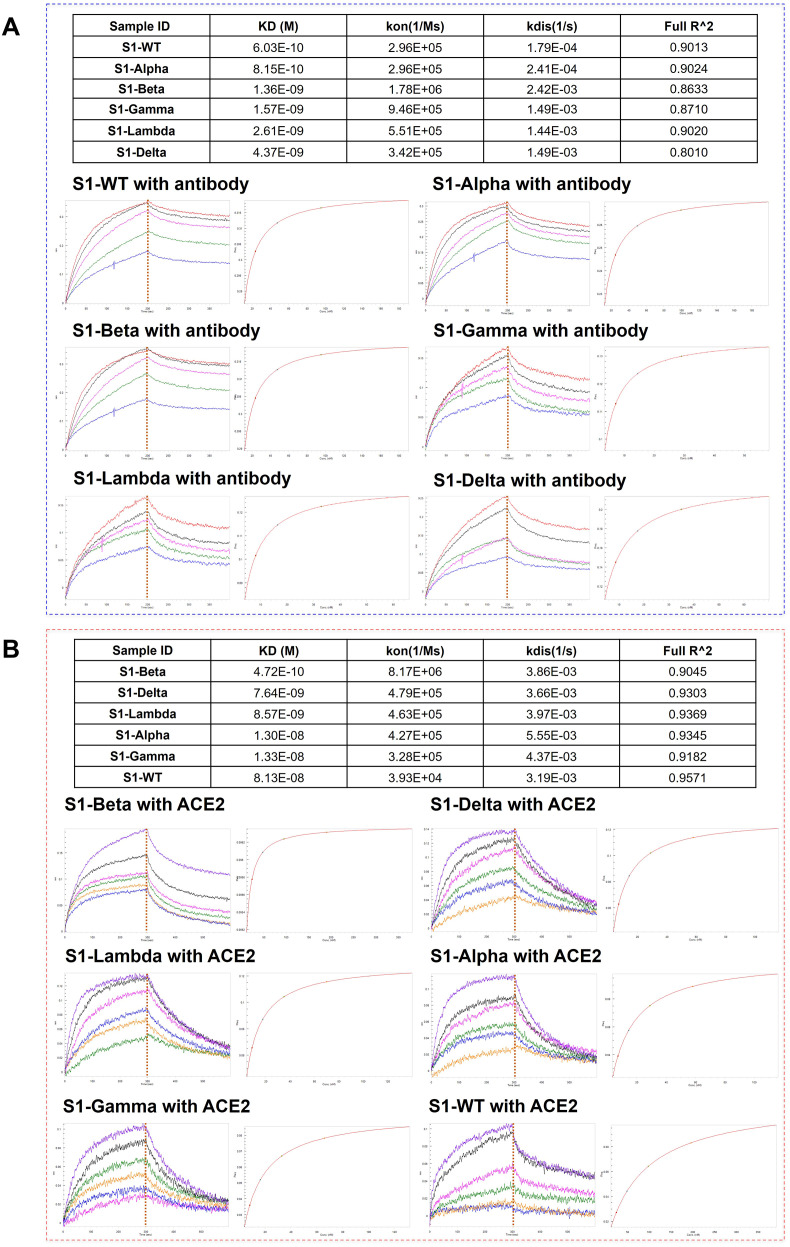
Comparison of the binding affinity of S1-antibody and ACE2 to different S1 proteins. **(A)** The binding ability of S1-antibody to the recombinant SARS-CoV-2 S1 proteins from WT and its variants measured by BLI. **(B)** The binding ability of ACE2 to the recombinant SARS-CoV-2 S1 proteins from WT and its variants measured by BLI. The association rate constants (Kon) and dissociation rate constants (Kdis) were determined by global fitting of the experimental data using a 1:1 binding model. Equilibrium dissociation constants (KD) were obtained from Kdis/Kon.

To further elucidate the correlation between N-glycosylation structure and S1 protein function, we performed BLI assays to explore the binding affinity of S1-antibody and ACE2 to recombinant S1 protein from WT and other variants. BLI provides reliable kinetics information, including the association rate (kon), dissociation rate (kdis), and equilibrium dissociation constants (KD) for protein-protein interactions. The results of the BLI experiments are presented in **Figure 7**. On one hand, S1-antibody binds to all the S1 proteins with overall good affinity values (10^-10^ < KD < 10^-9^) as shown in **Figure 7**. The KD value of the interaction between S1-WT and S1-antibody was 0.603 nM, R^2^ = 0.9013 (Steady state analysis, right panel), which confirmed that S1-antibody targets and tightly binds to S1-WT. The binding affinity of WT-S1 protein with S1-antibody was higher than that of the variants (S1-WT> S1-Alpha> S1-Beta> S1-Gamma > S1-Lambda > S1-Delta, **Figure 7A**). On the other hand, the results of the binding kinetics and affinity analysis of all S1 proteins interacting with ACE2 are shown in **Figure 7B**. We found that the KD value for ACE2 binding to the S1-Beta (0.472 nM) was around 172-fold lower than that of the S1-WT (81.3 nM) (**Figure 7B**). The S1 proteins of Beta, Delta, and Lambda variants displayed higher affinities to ACE2 than Alpha, Gamma, and WT (S1-Beta> S1-Delta> S1-Lambda> S1-Alpha > S1-Gamma > S1-WT, **Figure 7B**).

## 4 Discussion

Viral glycosylation is critical in the virus’s lifecycle, impacting stability and infection. The complex “glycan shield” on the SARS-CoV-2 spike protein is crucial to modulates protein folding, conformational stability, and immune evasion, making it essential to understand its glycosylation for vaccine and serological studies. As the virus mutates, how these mutations affect glycosylation remains a question worth studying. This study analyzes the glycosylation profiles of WT and five variants S1 proteins expressed in HEK293 cells, focusing on site-specific glycosylation and N-glycans characterization in recombinant spike proteins to better understand the similarities and differences of the “glycans shield” on virus variants, as well as the binding abilities of different variants to ACE2 and antibodies.

By enhancing the sensitivity of glycan detection and expanding the scope of glycan analysis, this research delves deeper into the structural and modification patterns of N-glycans. It also broadens the exploration of uniquely modified glycans, such as those with distinct sialic acid linkages (α-2,3 and α-2,6) and acidic glycans with acetylation modifications. These advancements enable a more comprehensive understanding of the glycosylation profile of N-glycans on the S1 protein. The distribution of 180 N-glycan compositions in the S1 and RBD regions of the S1 protein was investigated, revealing distinct glycosylation profiles across different areas. In the Alpha variant, the S1 region exhibited the highest fucosylation level among the WT and five variants, while the RBD region showed the lowest fucosylation level, presenting a striking contrast. Similarly, the Delta variant displayed region-specific differences in sialylation. S1-Delta displayed the lowest sialylation level among the WT and five variants, while the high sialylation in the RBD region was exhibited.

Acetylated glycans also demonstrated regional distribution patterns: acetylated glycans in the S1 region were consistently modified with fucosylation, whereas those in the RBD region exhibited a more complex multi-sialylation pattern. In terms of quantity, unlike the distribution patterns of N-glycans and glycopeptides, acetylated glycans were not concentrated in the RBD region but instead accumulated more prominently in the S1 region. Acetylated glycans modify the conformation of viral surface glycoproteins, influencing their binding efficiency to host cell receptors. As such, further investigation into acetylated modifications contributes to a more complete understanding of the glycosylation profile. The α-2,3 and α-2,6 linkages were critical determinants for the cross-species transmission of viruses (47, 48). An interesting observation was made by comparing the distribution of sialylated glycans between WT and various variants. Although the Lambda variant has a lower proportion of acidic glycans in the S1 region than other variants, the proportion of sialylated glycans in the RBD region of the Lambda variant is higher than the other five variants (**Figure 1**). This includes a significantly higher proportion of di-sialylated (25.3%) and multi-sialylated glycans (25.3%), which represent the majority of glycans in the Lambda RBD region. Moreover, the Lambda variant shows an increased number and more complex structures of α-2,3 and α-2,6 linked acidic glycans in the RBD region, further supporting this conclusion. These glycosylation profiles (**Supplementary Table S7**) indicate that the Lambda variant may have a stronger “glycan shielding effect” in the RBD region than others.

More than half of the glycosylation sites in the S1 protein are located in the N-terminal domain, with mutation sites primarily positioned near these glycosylation sites. Notably, the S1-Delta mutations T19R and the S1-Lambda mutations T76I eliminate N-glycosylation sequons at N17 and N74, respectively, which could potentially alter the glycosylation profile compared to the WT. S1-Delta exhibits fewer sialylated glycans and mono-fucose, while the lower levels of glycosylation modifications largely characterize the S1-Lambda variant. This suggests that losing N17 and N74 glycosylation sites might reduce modifications and decrease N-glycan diversity in S1 regions (**Supplementary Table S7**). The L452Q mutation in the Lambda variant significantly enhances RBD-ACE2 affinity, suggesting a potential mechanism for its increased binding to ACE2 (49). BLI assays demonstrated that the Lambda variant has a higher binding affinity with the ACE2 receptor than the WT (**Figure 7B**), probably resulting from reduced binding free energy and increased hydrogen bonding in the RBD-ACE2 complex (50). Mutations in the amino acids of the S1 protein significantly contribute to immune escape in SARS-CoV-2 variants, such as the Delta variant. These mutations may affect the neutralizing capacity of the S1 antibody against the RBD epitopes on the variant. For example, mutations such as E484K, T478K, S371L, N440K, and E484A found in the Beta, Delta, and Omicron variants may affect the ACE2 binding and neutralizing ability of antibody, which in turn could impact the immune response to these variants (51–53). The S1 antibody binding assay further supports this, showing reduced binding affinity for the Beta and Delta variant compared to the WT (**Figure 7**). Complex antigen epitopes and the formation of large immune complexes through antibody-antigen interactions may also be involved (54). Further, unoccupied N-glycosylation sites were observed in S1-Gamma (N657), S1-Delta (N61), and S1-Lambda (N17, N61, and N657), particularly at N17 and N61 with lower N-glycans occupancy (0% - 3.4%), and that these sites may lack glycan shield protection. Another mutation worth studying is D614G, which is a well-conserved substitution presented by SARS-CoV-2 variants and enhances viral fitness and immune evasion by modulating glycosylation at the nearby N616 site. In this study, there was a rise in tri- and tetra-antennary N-glycans at N616 compared to the WT, as well as an increased number of N-glycans (**Figures 4**, **6**). Variations in glycosylation patterns, particularly in the RBD region, impact the structural stability, infectivity, and ACE2 binding affinity of the spike protein. (15, 55–57). This study showed notable glycosylation variation among WT and different variants. In addition, BLI experiments suggested substantially varied ACE2 binding capacities across WT and five variants. However, this evidence is insufficient to support the role of glycosylation in ACE2. Previous studies have shown that amino acid mutations (such as D614G, N501Y, E484K, and L452R) are the critical factor for ACE2 affinity and immune evasion of SARS-CoV (58, 59). Lan et al’s research further suggested that specific residues in the SARS-CoV-2 RBD motif, such as Tyr449, Gly496, and Asn501, interact directly with ACE2 residues (Lys31, Glu35, and Asp38) (6). Hence, further research is necessary to confirm whether glycosylation serves as a functional factor independent of amino acid mutations.

The glycosylation profile of viral glycoproteins can vary across different host cells, affecting their interaction with the ACE2 receptor. In this study, all variants of the recombinant S1 protein and ACE2 were expressed in human HEK293 cells, ensuring comparability between different variants. HEK293 cells are a commonly used human-derived expression system in glycosylation and COVID-19 vaccine research, and the glycosylation patterns might be comparable to those in humans, albeit with some limitations. While HEK293 cells can perform complete glycosylation modifications, significant differences in glycan types, structures, and branching patterns may exist when compared to other host cells, such as CHO cells or non-mammalian cells (60, 61). These differences could influence protein stability, function, and interactions, particularly the binding affinity between ACE2 and the S1 protein. Therefore, careful selection of host cell lines is essential when studying the function and glycosylation of the SARS-CoV-2 spike glycoprotein to avoid potential biases. Moreover, it is important to note that the S2 domain of the spike protein also contains several glycosylation sites (e.g., N709, N717, N801, N1074, N1098, N1134, N1158, N1173, and N1194). The absence of the S2 domain may alter protein trafficking, potentially impacting the glycosylation profile of the spike protein (62). Therefore, further research on the glycosylation of the S2 domain would contribute to a more comprehensive understanding of the glycosylation patterns across the entire spike protein in different variants.

## 5 Conclusions

We comprehensively analyzed the glycosylation profiles of the S1 and RBD regions of the S1 protein across WT and five distinct variants expressed in HEK293 cells. The research reveals significant regional differences in glycosylation, particularly in the different regions. Notably, S1-Alpha showed opposing trends in fucosylation across S1 and RBD regions, while the Delta and Lambda variant exhibits regional differences in sialylation. Especially the Lambda variants show an increased number and more complex structures of α-2,3 and α-2,6 linked acidic glycans in the RBD region.

This study focuses on acetylated glycosylation on different variants for the first time. A key finding is that acetylated glycans are concentrated in the S1 region rather than the RBD region, contrasting with the distribution of N-glycans. In addition, amino acid mutations in the S1 protein of different variants, which lead to the elimination and mutation of glycosylation sites, reduce the diversity of N-glycans and cause significant changes in the glycosylation profile. These findings provide insights into the glycosylation differences of SARS-CoV-2 variants and support the construction of detailed glycosylation profiles for each variant, combined with the differences in binding affinities of the variants to ACE2 and antibodies, laying the foundation for future research on their functional correlations with the virus. Especially in the RBD region, which requires further investigation. Such discoveries offer valuable implications for subunit vaccine development and the study of COVID-19 transmission and treatment.

## Data Availability

The original contributions presented in the study are included in the article/**Supplementary Material**. Further inquiries can be directed to the corresponding authors.

## Supplementary material

Supplementary Figure 1 Intact N-glycopeptides were obtained by digestion of recombinant SARS-CoV-2 S1 protein with two complementary proteases. The amino acid sequence of S1 protein and potential N-glycosites and enzyme cleavage sites were displayed.
