# Supplementary figures and images for: Comprehensive N-glycosylation profiling of recombinant spike S1 protein from the wild-type SARS-CoV-2 and its variants
Source: Front Immunol. 2025 Jul 16;16:1592142. doi: 10.3389/fimmu.2025.1592142 (PMC12314560; doi:10.3389/fimmu.2025.1592142)

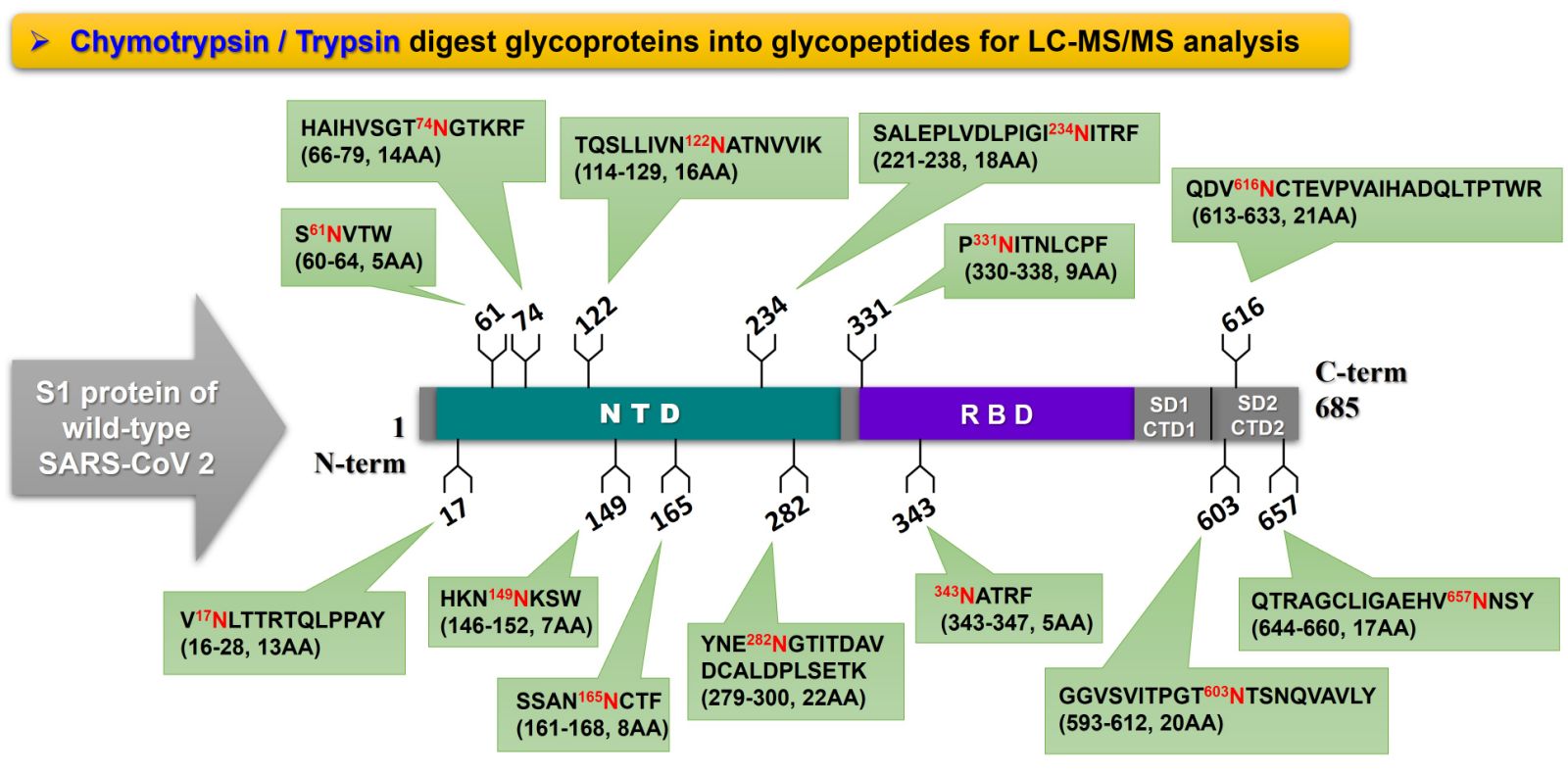

Supplement: Supplementary Figure 1 — Intact N-glycopeptides were obtained by digestion of recombinant SARS-CoV-2 S1 protein with two complementary proteases. The amino acid sequence of S1 protein and potential N-glycosites and enzyme cleavage sites were displayed. [file Image1.jpg]
